# Dispersion Engineering and Sensitivity Enhancement in Photonic Crystal Waveguide Sensors: Current Advances and Emerging Challenges

**DOI:** 10.3390/s26061872

**Published:** 2026-03-16

**Authors:** Nikolay L. Kazanskiy, Nikita V. Golovastikov, Svetlana N. Khonina

**Affiliations:** 1 Image Processing Systems Institute, NRC “Kurchatov Institute”, 151 Molodogvardeyskaya, Samara 443001, Russia; golovastikov.nv@ssau.ru (N.V.G.); khonina@ipsiras.ru (S.N.K.); 2 Samara National Research University, 34 Moskovskoye Shosse, Samara 443086, Russia

**Keywords:** photonic crystal waveguide, dispersion engineering, sensor, hybrid material platform, slow light

## Abstract

Photonic crystal waveguides (PhCWs) have emerged as a leading platform for integrated optical sensing due to their ability to engineer dispersion, enhance light–matter interaction, and exploit slow-light effects. This review provides a comprehensive analysis of the fundamental physics, performance metrics, device architectures, and application domains that define the current state of PhCW-based sensing. Key mechanisms governing sensitivity, figure of merit, detection limit, and dynamic range are examined, with emphasis on the intrinsic trade-offs introduced by slow-light operation, including disorder-induced scattering, linewidth broadening, and thermal susceptibility. Advances in dispersion engineering, such as hole shifting, gentle confinement, and width modulation, are highlighted alongside novel architectures including slot PhCWs, hybrid material platforms, and plasmonic–photonic configurations. Their respective capabilities in enhancing analyte overlap, improving spectral stability, and expanding functional integration are critically assessed. Emerging applications in biochemical detection, environmental monitoring, and nanoscale particle sensing further illustrate the versatility of PhCWs within modern optofluidic and lab-on-chip systems. The review concludes by outlining key challenges and future directions, including disorder-resilient slow-light design, inverse-engineered structures, and platform-level integration, which collectively chart a path toward next-generation high-performance photonic crystal sensing technologies.

## 1. Introduction

Photonic crystal waveguides (PhCWs) have emerged as one of the most powerful platforms for integrated optical sensing due to their ability to engineer dispersion and confine light at subwavelength scales [1,2,3,4]. By introducing a line defect into a periodic photonic crystal lattice, these waveguides guide light within a photonic bandgap while offering strong field localization and enhanced light–matter interaction. This enhancement is particularly significant in the slow-light regime, where the group velocity is substantially reduced, and the effective interaction length between the guided mode and the surrounding medium is increased [5]. As a result, PhCW sensors have demonstrated exceptional sensitivities and have become promising candidates for biochemical, environmental, and refractive index sensing applications [2,6,7,8,9].

Despite the rapid progress of the past two decades, sensing performance in PhCWs is not solely determined by enhanced field confinement [10]. The engineered dispersion that enables slow light also increases susceptibility to fabrication-induced disorder, scattering losses, and thermal fluctuations [11,12]. These practical limitations influence the achievable group index, the linewidth of the transmitted signal, and ultimately the detection limit of the sensor [13,14]. In many cases, the theoretical sensitivity enhancement predicted by slow-light scaling cannot be fully realized because the associated propagation losses broaden spectral features and introduce noise that limits the minimum detectable wavelength shift [10,15]. Understanding the interplay between sensitivity, dispersion design, and loss mechanisms is therefore essential for reaching the true performance potential of these devices [16,17,18,19].

Recent advances in dispersion engineering have opened new pathways to address these challenges [20]. Techniques such as hole shifting, width modulation, and gentle confinement have enabled flatter slow-light bands and reduced disorder sensitivity [18,21]. New material platforms, including silicon nitride, polymers, and hybrid silicon–two-dimensional material architectures, expand the design space and introduce improved stability, reduced thermo-optic drift, and additional surface functionalization options [22,23]. At the same time, integration with microfluidics and complementary photonic components has progressed, enabling the development of compact, robust, and application-specific lab-on-chip sensing systems [24,25].

Nevertheless, fundamental design trade-offs remain, particularly in balancing strong slow-light enhancement with low propagation loss and stable operation [26,27]. The detection limit of PhCW sensors is influenced not only by intrinsic device properties but also by external factors such as laser noise, temperature drift, and measurement modalities [28]. As applications such as clinical diagnostics, environmental monitoring, and single-particle detection demand increasingly stringent performance metrics, a comprehensive reassessment of the governing physics, engineering strategies, and practical limitations is needed [29,30]. Although PhCWs have demonstrated remarkable sensitivity enhancements through slow-light engineering and strong light–matter interaction, their advantages are best understood when viewed in the broader context of integrated photonic sensing platforms. Conventional silicon waveguides, slot waveguides, microring resonators, plasmonic structures, and nanobeam cavities each offer distinct strengths and weaknesses in terms of field confinement, dispersion control, propagation loss, and sensing performance. A direct comparison is therefore essential to motivate the unique role of PhCWs in advanced on-chip sensing [31].

To provide a clear perspective on these differences, Table 1 summarizes the fundamental characteristics, advantages, and limitations of PhCWs in comparison with other integrated waveguide architectures. This comparison illustrates how the combination of dispersion-engineering flexibility, slow-light enhancement, and strong analyte overlap sets PhCWs apart from competing platforms and motivates a deeper examination of their sensing behavior. Building on this foundation, Section 2 discusses the fundamental physics that govern dispersion, confinement, and loss. Section 3 analyzes the key sensing metrics that determine practical performance. Section 4 reviews major device architectures and engineering strategies, followed by Section 5, which outlines established and emerging application domains. Section 6 addresses the practical constraints that limit real-world operation, and Section 7 highlights future research directions that may enable the next generation of high-performance photonic crystal waveguide sensors.

## 2. Fundamental Physics of PhCWs

PhCWs derive their exceptional sensing capabilities from their unique dispersion characteristics and the ability to confine and manipulate electromagnetic energy on the scale of the wavelength. Unlike conventional dielectric waveguides, PhCWs operate through the presence of a photonic bandgap that prevents light from propagating in certain directions. By introducing a line defect within the periodic lattice, a guided Bloch mode is formed inside this bandgap, allowing tight optical confinement and highly tunable dispersion [49]. These properties make PhCWs particularly suitable for applications that require strong slow light enhancement, large analyte–field overlap, and precise control over the sensor response. The following subsections describe in detail the structural dispersion, light–matter interaction, and loss mechanisms that ultimately determine the performance of PhCW sensors.

### 2.1. Structure and Dispersion Characteristics

Two-dimensional PhCWs are commonly realized by removing a single row of air holes or dielectric rods from an otherwise periodic lattice [50,51]. This modification creates what is known as a W1 waveguide in SOI platforms, although numerous variations exist. The line defect supports localized photonic states whose dispersion can be shaped through adjustments to the lattice constant, the radii of nearby holes, the positions of the first and second rows of holes, or even the use of noncircular hole geometries, such as elliptical apertures. These design parameters directly influence the dispersion relation between angular frequency and wavevector. Close to the Brillouin-zone boundary, the dispersion curve naturally flattens, producing a regime of slow light where the group velocity becomes significantly reduced [52]. In the slow-light regime, the group index can increase to very high values, often exceeding several tens or even surpassing one hundred in carefully engineered structures [53]. The increase in group index enhances the interaction time between the optical mode and the surrounding medium, which is crucial for high-sensitivity sensing. However, a large group index also narrows the usable bandwidth and elevates the influence of fabrication-induced irregularities [54]. To mitigate these issues, modern design strategies make use of hole-shifting and gentle confinement techniques that smooth the modal profile and reduce group-velocity dispersion. Elliptical-hole PhCWs, for example, have shown that by appropriately orienting the elliptical holes and applying longitudinal shifts to selected hole rows, it is possible to achieve flat slow-light bands with reduced dispersion while maintaining large and nearly constant group indices [55].

From a broader wave-physics perspective, slow-light phenomena can also arise in resonant media composed of two-level systems, where pulse propagation is governed by coherent light–matter interaction, as described by McCall and Hahn in the context of self-induced transparency [56]. In such systems, the propagation medium can influence pulse characteristics such as pulse area, central frequency, and temporal duration. More recent studies on quantum metamaterials have further explored how structural inhomogeneity in active elements enables additional pulse-control mechanisms [57]. Although PhCWs operate in the classical dielectric regime rather than through resonant two-level interactions, an analogous conceptual role is played by photonic band-structure engineering. In PhCWs, the periodic refractive-index modulation and controlled structural perturbations reshape the dispersion relation and density of optical states, thereby governing group velocity, phase accumulation, and pulse broadening. Thus, while the underlying physical mechanisms differ, both systems illustrate how engineered medium structure can determine wave propagation properties.

Ding et al. reported one-dimensional and two-dimensional PhC cavities created in thin diamond membranes [58]. The devices reached Q-factors of approximately 1.8 × 10^5^ and 1.6 × 10^5^, which are among the highest values recorded for PhC cavities operating in the visible spectrum. An important strength of their method is that the photonic structures are fabricated through simple planar processes with high yield, avoiding the complicated undercut steps required in earlier approaches. They also demonstrated efficient fiber coupling to a one-dimensional cavity and observed clear interaction between a single silicon vacancy center and the cavity mode at a temperature of four kelvin, accompanied by a Purcell enhancement of eighteen.

**Figure 1:**
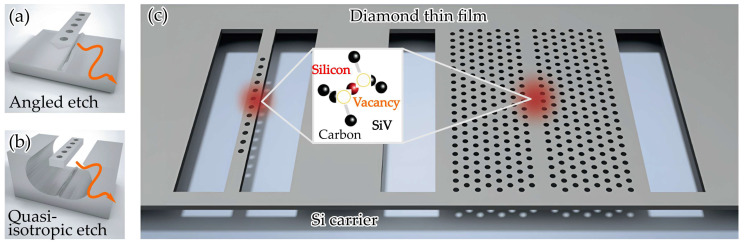
(**a**) Angled etching of a one dimensional PhC cavity, which produces a triangular cross section and limits the use of dense patterns because the ion beam grazes nearby features; (**b**) quasi isotropic oxygen etching used to release a one dimensional cavity, although the long undercut time and the resulting bottom surface roughness remain major drawbacks; (**c**) thin film fabrication where the cavity is defined by top down etching and the undercut is applied only to the substrate. This method avoids bottom roughness, simplifies processing, and enables much higher quality factors and more flexible photonic designs [58].

Diamond PhC devices have traditionally been produced using several bulk machining techniques. Angled etching, including Faraday cage and ion-based angled etching, remains one of the most common approaches and is illustrated in Figure 1a. These techniques generate suspended nanobeams but often leave noticeable roughness because the undercutting step is difficult to control. Quasi-isotropic etching, represented in Figure 1b, releases the nanobeam using an oxygen-based isotropic process, although the long etch times and the resulting rough lower surfaces remain significant disadvantages. As a result, both methods typically produce photonic crystal cavities with quality factors in the low ten thousand range, far below the theoretical values predicted by simulations. Thin-film fabrication methods were introduced to avoid these limitations by eliminating the need for a bulk undercut. Earlier thin film processes, however, were limited by thickness nonuniformity and reduced diamond crystal quality, which prevented high cavity performance. These issues motivate the thin film platform shown in Figure 1c, which offers improved fabrication control while preserving high-quality diamond, enabling a more capable foundation for advanced photonic devices [58].

### 2.2. Enhanced Light–Matter Interaction

The sensitivity of PhCW sensors is closely tied to the degree of light–matter interaction that takes place within the device [59]. When the waveguide operates in the slow-light regime, the optical mode propagates with reduced velocity, effectively increasing the duration of its interaction with the analyte. This extended interaction time greatly amplifies the effect of even small refractive-index perturbations. In addition to slow-light enhancement, the spatial field distribution within the waveguide plays a central role. The electric field often becomes concentrated near the first row of holes adjacent to the defect, which is precisely where analytes are introduced through infiltration or microfluidic delivery [60]. By modifying the lattice geometry, the field can be intentionally pushed deeper into the sensing region, increasing the modal overlap and strengthening the sensitivity to refractive-index variations [61,62].

Yashaswini et al. developed a highly responsive 1D PhC–based sensor for chemical detection [63]. Four pairs of materials were explored for the alternating layers: magnesium fluoride/cadmium fluoride, tantalum pentoxide/silicon dioxide, titanium dioxide/silicon dioxide, and zinc sulphide/silicon dioxide. The defect layer thickness was varied between 3500 nm and 5000 nm. Simulation results revealed that all material pairs provided sensitivities exceeding 500 nm/RIU. Among them, the titanium dioxide–silicon dioxide structure demonstrated the greatest sensitivity, reaching 675 nm/RIU. When the defect layer thickness surpassed 4000 nm, the transmission efficiency approached 98%. The proposed sensor achieved a figure of merit (FOM) of 8437, a detection limit of 7.30 × 10^−6^ RIU, and a maximum quality factor of 13,687 [63].

**Figure 2:**
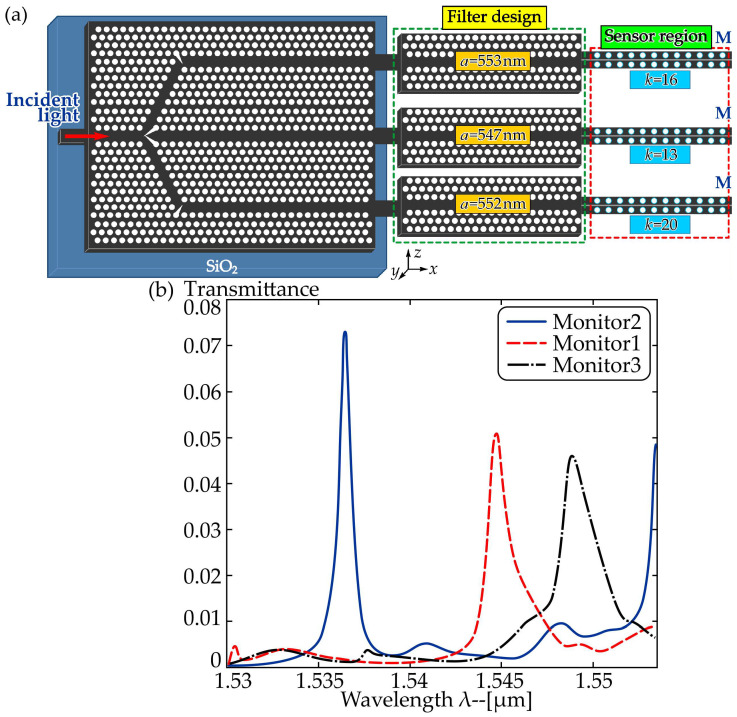
(**a**) Schematic of the PhC parallel-integrated sensor array implemented on a monolithic substrate. The design incorporates a 1 × 3 beam splitter and three single-slot PhCNC sensing units arranged in parallel. (**b**) Simulated transmission spectra of the three parallel branches, each containing an individual PhC sensor. Three distinct resonant peaks are observed, corresponding to the three parallel sensing channels [64].

Under the framework of enhanced light-matter interaction in PhC structures, Zhou et al. developed an efficient simulation approach for a sensor array based on high-sensitivity single-slot photonic crystal nanobeam cavities (PhCNCs) integrated on a silicon platform [64]. By combining a carefully engineered PhCW filter with an optimized single-slot PhCNC, a specific high-order resonant mode was effectively isolated for sensing. A 1 × 3 beam splitter was implemented to divide the optical signal and integrate three sensing units, enabling a compact microarray configuration. Multiplexing is realized by placing tailored resonant cavities at the ends of each beam-splitter branch, producing distinct resonant peaks for each channel. This arrangement allows multiple cavity sensors to be interrogated simultaneously on a single monolithic PhC device. To validate the multiplexing strategy and the performance of the PhCW filters, a three-channel PhC sensor array was designed. The 1 × 3 PhC beam splitter distributes light into three branches, where the engineered passbands enable integration of three high-sensitivity multimode single-slot PCNC sensors, as shown in Figure 2.

In each channel, the PhCW filter functions as a wavelength selector, while the PCNC acts as the sensing element through its cascaded transmission band. Careful design of the PhCW filters ensures clear separation of the targeted high-order resonant modes, allowing all sensors to be read simultaneously from the combined transmission spectrum. Each sensor can accommodate a different analyte. Based on the integrated structure in Figure 2a, 3D-FDTD simulations were performed, and the resulting output spectra are shown in Figure 2b. As expected, three well-defined resonant peaks appear in the transmission response. Three-dimensional finite-difference time-domain (3D-FDTD) simulations yielded sensitivities of S1 = 492 nm/RIU, S2 = 244 nm/RIU, and S3 = 552 nm/RIU.

### 2.3. Loss Mechanisms in PhCWs

Although slow light improves sensitivity, it also significantly increases propagation loss. One of the most critical sources of loss in PhCWs is disorder-induced scattering. Fabrication imperfections such as hole-position deviations, sidewall roughness, variations in hole radius, and nonuniform etching introduce random perturbations that scatter light out of the guided mode. The severity of this scattering increases rapidly as the group index becomes larger because slow-light modes exhibit greater spatial localization and stronger sensitivity to small geometric deviations. These disorder effects can distort the Bloch mode, introduce random phase fluctuations, and broaden the spectral features that are essential for precise sensing.

Another important loss mechanism arises from vertical radiation leakage. In slab-type photonic crystals, vertical confinement relies on total internal reflection. Variations in geometry or in the modal distribution can couple energy into radiation modes that leak into the substrate or air cladding. This effect becomes particularly problematic near the band edge, where the optical field contains stronger high-spatial-frequency components that approach the light cone. Designs that distribute the optical field more smoothly across the unit cell tend to exhibit reduced radiation loss and improved spectral stability. The interaction between sensitivity and loss creates an unavoidable trade-off. While increasing the group index enhances the sensitivity of the device, it simultaneously introduces higher propagation losses, broader resonance linewidths, reduced signal-to-noise ratio, and ultimately higher detection limits. These coupled effects highlight the importance of careful dispersion engineering in PhCW sensors.

### 2.4. Interplay Between Dispersion, Loss, and Sensitivity

The physical mechanisms described above do not act independently. Instead, they form a tightly connected set of trade-offs that defines the practical performance window of PhCWs. Enhancing the group index increases the sensitivity by magnifying light–matter interaction. However, this same enhancement also increases scattering losses and broadens the spectral linewidth, which directly degrades the achievable figure of merit and raises the limit of detection. Thermal sensitivity and environmental fluctuations further complicate operation at very high group indices. As the dispersion curve becomes steeper near the band edge, nonlinearities emerge, reducing the dynamic range and causing the wavelength shift to deviate from a linear function of refractive-index change.

These interconnected effects make dispersion engineering an optimization challenge rather than a single-parameter problem. The best-performing PhCW sensors do not maximize the group index but instead maintain a balanced operating point where sensitivity, linewidth, loss, and spectral stability reach a practical equilibrium. Advanced structures such as width-modulated waveguides, elliptically perturbed lattices, slot-inserted PhCWs, and hybrid material systems are all attempts to shift this equilibrium toward higher practical performance. The interplay between dispersion, loss, and sensitivity thus forms the fundamental physical basis upon which all subsequent sensing metrics and device architectures are built.

## 3. Key Performance Metrics

### 3.1. Sensitivity

Sensitivity in PhCW sensors quantifies how effectively the optical mode responds to changes in the refractive index of either the bulk surrounding medium or surface-bound analytes [51,64,65]. Bulk sensitivity, expressed in nm/RIU, measures the resonance or transmission-wavelength shift per unit refractive index change and is strongly enhanced by the slow-light effect, where the group index increases the interaction time between light and analyte. Surface sensitivity, expressed in pm/nm, characterizes the wavelength shift per nanometer of surface-layer adsorption and depends heavily on the spatial localization of the electric field, particularly near the first row of air holes or slot regions in modified PhCW geometries [66]. Both quantities scale with the optical mode overlap factor, which depends on field penetration into the sensing region, as well as the effective modal confinement governed by the geometry of the PhC lattice.

The group index plays a central role, as higher values enhance perturbation-induced wavelength shifts according to first-order electromagnetic perturbation theory [67]. However, stronger confinement within the high-index core can reduce effective overlap with the analyte, while more delocalized modes near the band edge produce higher overlap but are more susceptible to disorder-induced scattering. When compared to competing sensing platforms, PhCWs typically exceed the bulk sensitivity of microring resonators due to the absence of a strict resonant energy-storage condition and their ability to maintain broadband operation with engineered dispersion [68]. PhCWs offer higher design flexibility than slot waveguides while avoiding the extreme fabrication sensitivity inherent to nanometer-scale slot gaps [36]. Relative to plasmonic sensors, PhCWs offer lower intrinsic losses, larger propagation lengths, and improved CMOS compatibility, although plasmonic structures may still outperform in raw field confinement at metallic interfaces [69].

**Figure 3:**
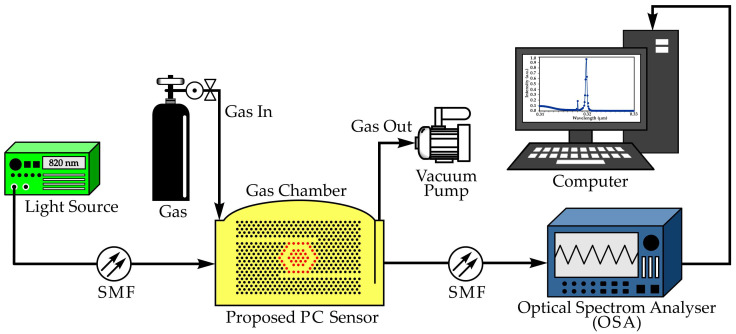
Experimental configuration of the gas sensor employing a 2D PhC structure [64].

Fallahi et al. examined the design and simulation of PhC micro-ring resonator optical sensors [64]. A symmetric micro-hexagonal ring resonator was selected, and several MHRR-based arrays were created to study how their arrangement influences the output spectrum. The performance of these configurations was compared with findings from other numerical studies. A clear design algorithm was presented to guide the development of PhC optical sensors and to identify the most effective structure. The role of reflector rods was also analyzed, showing a clear link between their spacing and the length of the MHRR in achieving an optimal design. The effects of changing the lattice constant and the radius of the dielectric rods on FWHM, transmission, and resonance wavelength were evaluated to determine the best operating mode. The optimized sensor was tested for gas detection and achieved 96% transmission, 0.31 nm FWHM, a Q-factor of 2636, a sensitivity of 6451 nm/RIU, and a FOM of 2960 RIU^−1^ [64]. The experimental configuration of the gas sensor employing a 2D PhC structure is shown in Figure 3.

In another study, the authors explored the design and performed the simulation of optical sensors that utilize PhC micro-ring resonators (MRRs) [64]. Depending on the PhC configuration, a variety of waveguides and resonator geometries can be created, enabling multiple sensor topologies. Several arrangements of MHRR arrays were developed to examine how different layouts influence the output spectrum. The performance of these structural variations was analyzed and compared with previously reported numerical studies. A comprehensive and structured design algorithm was presented, addressing all critical aspects necessary for selecting optimal PhC-based sensor geometries. As part of this process, the influence of reflector rods was also evaluated, revealing a clear correlation between reflector-rod spacing and MHRR length in achieving an optimized configuration. Further analysis was conducted to determine how changes in lattice constant and dielectric-rod radius affect the full width at half maximum (FWHM), transmission, and resonant wavelength, enabling the identification of the most efficient operating mode.

To validate the performance of the optimized structure, its suitability for gas-sensing applications was assessed. The final sensor demonstrated a transmission of 96%, an FWHM of 0.31 nm, a quality factor (QF) of 2636, a sensitivity of 6451 nm/RIU, and a FOM of 2960 RIU^−1^. Comparisons with related works confirm that the design strategy adopted here offered a systematic and effective pathway for generating and evaluating diverse MRR-based photonic crystal sensor topologies. The methodology avoids arbitrary parameter tuning and streamlines the optimization process, making it a practical foundation for developing PhC-MRR devices intended for optical integrated circuit applications [64].

### 3.2. Figure of Merit (FOM)

The spectral FOM is defined as the ratio between the sensitivity and the full-width-at-half-maximum (FWHM) spectral linewidth of the transmission feature, reflecting the device’s ability to resolve small refractive-index-induced shifts [70]. In slow-light PhCWs, the FOM strongly depends on the group index because linewidth broadening arises from increased scattering and material absorption at high ng. As the group index increases, both the sensitivity and the attenuation coefficient scale upward, leading to a reduction in the sharpness of spectral features. The spectral linewidth broadens approximately in proportion to total propagation loss, which increases rapidly near the band edge due to enhanced localization of the Bloch mode and the breakdown of idealized Bloch-wave transport in the presence of fabrication disorder [71].

Thus, while the sensitivity may scale linearly with group index, the denominator of the FOM, which is the optical linewidth, typically grows nonlinearly with loss, resulting in a practical optimum group-index range that maximizes FOM. Excessive slow-light operation reduces the usable dynamic range and diminishes contrast in spectral transduction [72]. Optimized PhCW designs, including width-modulated and gently confined architectures, mitigate these issues by flattening the dispersion curve and reducing the group-velocity dispersion, thereby enabling improved FOM performance compared to conventional W1 waveguides or bulk silicon waveguide sensors. An optical sensing platform was introduced that employed a high-Q slot PhC cavity filled with various test liquids, all implemented on a supported membrane rather than a suspended one [3]. When the cavity was infused with liquids spanning refractive indices from 1.345 to 1.545, the device exhibited a wavelength shift sensitivity of 235 nm/RIU. Throughout these measurements, the cavity’s Q-factor remains between 8000 and 25,000, leading to a FOM that can reach approximately 3700. The sensor could detect refractive-index changes as small as 1.25 × 10^−5^. Built on a silicon-on-insulator platform, the design ensures mechanical robustness and facilitates seamless integration into existing silicon photonic technologies [3].

**Figure 4:**
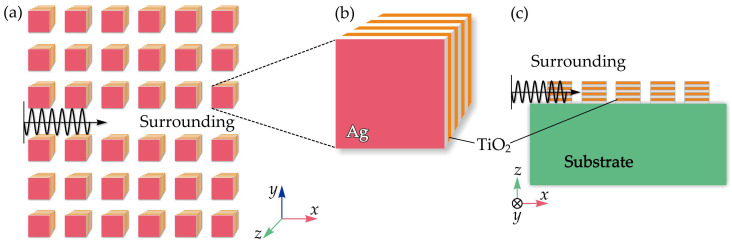
Schematic diagrams of (**a**) the HMM–PhC waveguide and (**b**) the HMM nanorod formed from alternating Ag and TiO_2_ layers; (**c**) cross-sectional view of the HMM–PhC waveguide [73].

Recently, Zheng et al. introduced a compact sensing platform that integrates hyperbolic metamaterials (HMMs) with PhCs, two structures with distinct dispersion properties that enable strong control over light propagation [73]. The proposed PhC waveguide, formed from arrays of HMM nanorods, supports direct coupling between plasmonic and guided modes, allowing the system to function as a high-FOM nanosensor (Figure 4a–c). Changes in the refractive index of the surrounding medium lead to measurable shifts in absorption and transmission, enabling the detection of temperature, pressure, or material variations. By selecting appropriate surrounding materials, for example, thermally sensitive infrared media, the waveguide response can be tailored to different sensing needs. Full electromagnetic simulations based on the finite element method were used to evaluate device performance. The results show a sensitivity of 324.16 nm/RIU and an FOM of 469.58 RIU^−1^, representing a significant improvement over conventional plasmonic sensors. This enhancement is primarily due to the strong plasmonic–waveguide mode interaction enabled by the HMM-PhC architecture. Although fabrication may be challenging due to the system’s complexity, ongoing advances in nanofabrication are expected to mitigate these issues. With its high performance and versatility, the HMM-PhC waveguide sensor holds strong potential for applications in biomedical diagnostics, environmental monitoring, and other photonic sensing fields [73].

### 3.3. Limit of Detection (LOD)

The limit of detection represents the minimum refractive-index or surface-induced change that can be reliably resolved and is governed by the noise-equivalent wavelength shift (NEWS), which sets the smallest detectable departure from baseline spectral position [74]. NEWS arises from multiple sources, including propagation loss, laser frequency resolution, thermal fluctuations, and fabrication disorder [75]. Device loss broadens the spectral response and reduces contrast, thereby decreasing the ability to detect small perturbations. Laser resolution imposes a hard lower bound on spectral discrimination, especially for interrogation schemes using tunable diode lasers or swept-source systems. Thermo-optic noise becomes particularly significant in silicon PhCWs due to the large thermo-optic coefficient and sensitivity of band-edge modes to temperature fluctuations [76].

Fabrication disorder imposes random spectral wandering and localizes Bloch modes in unpredictable ways, reducing coherence and increasing scattering-induced amplitude noise. These effects become pronounced in the deep slow-light regime, where even small deviations in hole position or sidewall roughness result in disproportionately large perturbations to the effective index. Consequently, the LOD is not solely determined by sensitivity but by the interplay between design-induced slow light, material stability, and measurement noise [77]. Optimized PhCW designs minimize these noise pathways by reducing disorder sensitivity and flattening the dispersion near the operational wavelength [78]. Tang et al. proposed a systematic optimization strategy for realizing slow-light behavior with high group index, broad bandwidth, and minimal dispersion in a PhCW featuring elliptical holes [78]. By adjusting the orientation of the first-row elliptical holes and applying a longitudinal shift to the holes in the third row, the group index, bandwidth, and dispersion can be effectively tailored. Under the condition that the group index remained within ±10% of its target value, the resulting flat-band bandwidths near 1550 nm were 13.5, 9.4, 7.8, and 6.1 nm for group indices of roughly 46, 63, 78, and 100, respectively. All configurations yield an almost unchanged group-index–bandwidth product of 0.39. Furthermore, low-dispersion slow-light performance was verified by examining temporal pulse broadening using two-dimensional finite-difference time-domain simulations [78].

### 3.4. Dynamic Range and Linearity

The dynamic range of a PhCW sensor defines the maximum analyte-induced refractive-index change or bound-layer thickness that can be accurately tracked before the device’s spectral response becomes nonlinear or saturates [79]. Near the photonic band edge, nonlinearities arise from the steep dispersion slope and high group-velocity dispersion, causing the wavelength shift to deviate from a linear relationship with refractive-index perturbation. These band-edge nonlinearities limit the usable operational window in slow-light-enhanced sensors [80].

Operating deeper into the slow-light regime increases sensitivity but reduces linearity, narrows the spectral window available for interrogation, and raises susceptibility to disorder-induced spectral distortions [81]. Thus, practical operation typically requires balancing slow-light enhancement against linear dynamic range constraints. Designs employing gentle confinement or modified lattice geometries improve linearity by reducing group-velocity dispersion while preserving elevated overlap factors. Ultimately, the dynamic range is bounded by the combined effects of dispersion nonlinearity, optical loss, and the onset of resonance distortion in the transmission spectrum [82].

Olyaee et al. introduced a redesigned PhC pressure sensor that offered high resolution along with a broad dynamic range [83]. The device was based on a two-dimensional PhC structure composed of a square lattice of silicon rods in an air background. Its architecture included a PhCW coupled to a nanocavity, where the waveguide is created by removing a single row of Si rods, and the nanocavity was produced by altering the radius of one rod. The sensor operated within the 1300–1400 nm wavelength window. Simulations indicate that the cavity’s resonant wavelength shifts linearly toward longer wavelengths as the applied pressure increases. The proposed design demonstrated linear response behavior from 0.1 GPa to 10 GPa and achieved a pressure sensitivity of approximately 8 nm/GPa [83].

### 3.5. Comparative and Critical Analysis of Performance Metrics

A holistic evaluation of PhCW sensor performance requires considering sensitivity, FOM, LOD, and dynamic range not as isolated metrics but as interdependent quantities shaped by the underlying dispersion, loss mechanisms, and fabrication constraints. Their relationships are largely governed by the group index, modal overlap with the analyte, and the degree of slow-light enhancement engineered into the photonic crystal band structure. Although increasing the group index generally improves raw sensitivity, the resulting increase in scattering loss and linewidth broadening simultaneously deteriorates FOM, restricts dynamic range, and ultimately limits the achievable LOD. Consequently, optimizing one metric often degrades another, revealing a fundamental set of trade-offs inherent to slow-light photonic sensors.

Sensitivity benefits most directly from increased overlap factors and slow-light enhancement, as perturbations in refractive index induce proportionally larger wavelength shifts near the band edge. However, high group-index operation intensifies the device’s susceptibility to fabrication disorder and modal localization, leading to larger spectral noise and broader resonance features, which reduce the practical resolution of the sensor. Thus, while theoretical sensitivity may increase monotonically with group index, the effective sensitivity defined as the smallest reliably resolvable shift is often constrained by disorder-induced scattering and thermo-optic fluctuations. This interplay between enhanced light–matter interaction and increased noise establishes an upper bound on useful slow-light operation.

The spectral FOM provides a clearer perspective on these trade-offs by incorporating linewidth broadening directly into the performance metric. As propagation loss increases in the slow-light regime, the spectral response becomes increasingly blurred, diminishing the advantage gained from higher sensitivity. An optimal operating region typically exists where the group index is high enough to improve sensitivity but low enough to avoid excessive scattering loss. Gentle-confinement strategies and band-flattening techniques attempt to extend this optimal window by reducing group-velocity dispersion and suppressing disorder sensitivity. When benchmarked against other platforms such as microring resonators or slot waveguides, PhCWs offer superior dispersion engineering flexibility, but their slow-light-induced linewidth degradation remains the primary factor limiting their maximum FOM.

These trade-offs directly influence the limit of detection. Since LOD scales with the noise-equivalent wavelength shift, any factor that increases spectral noise, such as temperature drift, laser instability, or disorder-induced localization, deteriorates the detection limit even if the intrinsic sensitivity remains high. In practice, LOD is therefore more closely correlated with linewidth stability and thermal noise than with sensitivity alone. This explains why PhCW sensors with similar sensitivities may exhibit vastly different detection limits depending on waveguide design, material platform, and environmental stability.

Dynamic range is similarly intertwined with slow-light dispersion and the onset of nonlinear spectral shifts near the band edge. As the perturbation magnitude increases, the linear relationship between analyte-induced refractive-index change and spectral response breaks down due to steep dispersion near the cutoff. Deep slow-light operation not only reduces dynamic range but also makes the sensor’s response increasingly nonuniform across the measurement window. Designs aiming for ultra-high sensitivity may therefore compromise the ability to perform measurements over a broad analyte concentration range. The optimal operating point thus requires a balance between sensitivity, linearity, and robustness to perturbations.

Collectively, these dependencies highlight that PhCWs do not achieve peak performance by maximizing a single metric, such as group index or modal confinement, but rather through a carefully engineered balance of dispersion flatness, low disorder sensitivity, controlled overlap, and stable thermal behavior. Unlike resonant sensors such as microrings or plasmonic nanoparticles, where high Q-factor or strong field confinement can be increased independently, PhCW sensors are governed by coupling mechanisms in which enhancements and degradations occur simultaneously. This interconnected nature underscores the need for system-level optimization approaches, including adjoint-based inverse design, hybrid material integration, and disorder-resilient lattice engineering, to push the realistic performance limits of slow-light photonic crystal sensing technologies. To facilitate a clearer comparison of how the principal performance metrics evolve under slow-light operation, Table 2 summarizes their dominant physical determinants and the key mechanisms that govern their improvement or degradation. Rather than reiterating the full analysis, the table distills the essential relationships among sensitivity, FOM, LOD, and dynamic range, highlighting how each metric is shaped by dispersion engineering choices and how changes in one parameter influence the others. This consolidated view provides a structural overview that complements the detailed discussion, enabling readers to quickly identify the central constraints and design trade-offs that define the operational window of PhCW sensors.

## 4. Device Architectures and Engineering Strategies

The diversity of PhCW geometries has led to a wide range of dispersion profiles, confinement behaviors, and sensing capabilities. Different architectural strategies, such as hole shifting, gentle confinement, width modulation, slot integration, and hybrid material incorporation, offer unique advantages in terms of slow-light bandwidth, field overlap, loss performance, and fabrication robustness. Table 3 summarizes the principal PhCW architectures reported in the literature, outlining their operating mechanisms, key benefits, and practical limitations. This comparison provides a foundation for the detailed discussion in the following subsections, where the impact of geometry and engineering techniques on sensing performance is examined.

### 4.1. Conventional W1 PhCWs

The W1 structure, obtained by removing a single row of holes from a two-dimensional PhC lattice, remains the foundational architecture for integrated PhCWs [102]. Its defect guided mode occupies the photonic bandgap and supports strong lateral confinement essential for sensing. The W1 platform is attractive because its geometry is simple, compatible with silicon photonic fabrication, and offers a predictable dispersion landscape that is straightforward to analyze and tune [103]. Engineering refinement in the W1 design most commonly involves adjusting the lattice constant and the sizes of the holes adjacent to the defect [104]. These modifications change the distribution of effective refractive index in the guiding region and alter the slope of the dispersion curve, enabling moderate control over the group index and bandwidth. Although the W1 structure does not inherently provide the high degree of slow light flattening achievable with more advanced approaches, it remains central to many sensing platforms due to its fabrication practicality and its suitability as a starting point for more sophisticated dispersion engineering methods [105].

Tanaka et al. conducted a theoretical investigation into the interaction between PhC cavities and waveguides in two-dimensional PhC structures [102]. Efficient energy transfer between a cavity and an adjacent waveguide is essential for the proper operation of integrated optical devices, making careful structural design crucial for achieving high performance. To establish practical design guidelines, they analyzed how the coupling strength varies as key geometric parameters are systematically modified, using finite-difference time-domain (FDTD) simulations. The resulting trends were interpreted in terms of the density of states of the waveguide bands and the symmetrical characteristics of both the cavity and waveguide modes. Their results revealed that the conventional parity-based selection rules can break down when mode linewidths broaden due to finite-time transitions [102].

**Figure 5:**
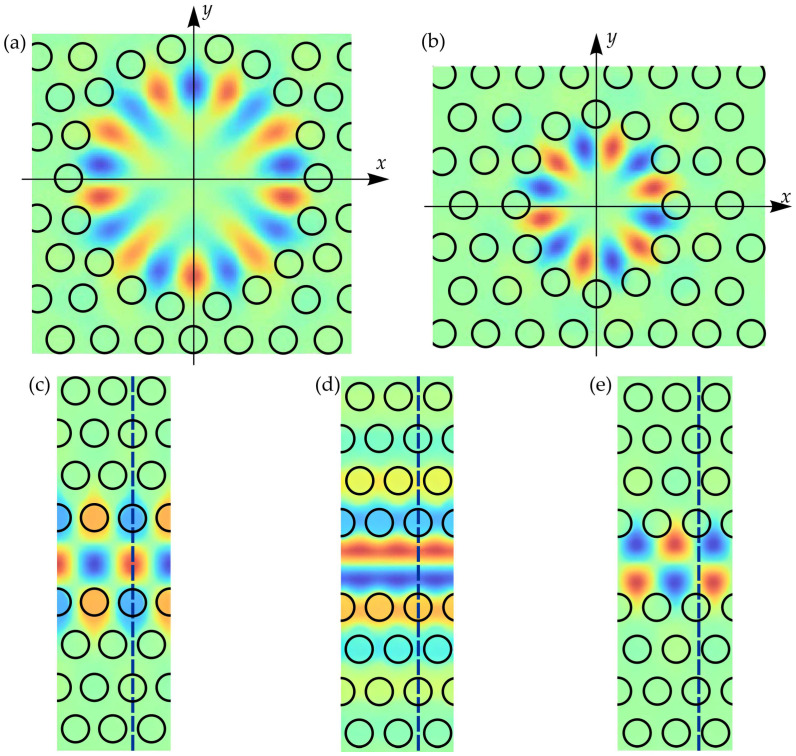
Spatial distribution of the electromagnetic field component *H_z_*(*x*,*y*) for the modes involved in the interaction between the cavity and the waveguide: (**a**,**b**) whispering-gallery modes of the modified H3 and H2 cavities, respectively; (**c–e**) the waveguide modes at the van Hove singularities. The dashed line marks the *y*-axis of the Cartesian coordinate system, whose origin is positioned at the center of the cavity. For panels (**c**–**e**), the dashed reference line corresponds to cases where *S* is an even integer. If *S* is odd, the *y*-axis should be shifted by *a*/2 [102].

Figure 5 presents the spatial distributions of the electromagnetic field *H_z_*(*x*,*y*) for the modes most relevant to cavity–waveguide coupling. Figure 5a and Figure 5b show the whispering-gallery modes of the modified H3 and H2 cavities, respectively. Among the various symmetry properties of these modes, mirror symmetry with respect to the y-axis plays a central role in determining the coupling strength. The whispering-gallery mode in the H3 cavity displays even symmetry with respect to this axis, whereas the corresponding mode in the H2 cavity exhibits odd (anti-symmetric) symmetry. Figure 5c–e illustrates the waveguide modes at the van Hove singularities: the X-point mode of band A, the Γ-point mode of band B, and the X-point mode of band B. As commonly noted in selection rule arguments, coupling between modes of opposite symmetry is typically weak [102].

### 4.2. Dispersion Engineering for Slow Light

More advanced device architectures exploit controlled geometric perturbations to reshape the dispersion relation with far greater precision than the baseline W1 design. A key strategy is the deliberate shifting of selected rows of holes along or perpendicular to the propagation direction. This controlled perturbation modifies the optical potential seen by the Bloch mode and enables the formation of a tailored slow light band. The resulting dispersion profile can be engineered to achieve higher group indices across a broader spectral range without requiring substantial changes to the global lattice. Another meaningful approach is gentle confinement. Instead of relying on a sharp defect boundary, gentle confinement gradually transitions into the photonic crystal lattice near the guiding region. This reduces abrupt refractive index variations and produces smoother modal profiles that are less sensitive to local geometric imperfections. Gentle confinement is particularly valuable in sensing architectures where reproducible spectral behavior is critical. Flat band slow light design further extends these concepts by minimizing group velocity dispersion around the operating wavelength. Through careful tuning of hole shapes or systematic width modulation of the waveguide, it becomes possible to maintain nearly constant group index across several nanometers of bandwidth. This enhances the reliability of wavelength shift measurements and preserves the linearity of sensor response, providing meaningful advantages in dynamic environments where analyte-induced perturbations vary over time.

**Figure 6:**
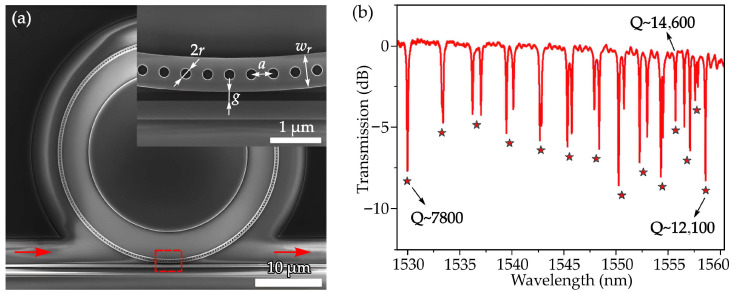
SEM image and measured transmission response. (**a**) SEM image of the fabricated air-mode PhCRR. The inset provides an enlarged view of the evanescent coupling section; (**b**) recorded TE-polarized transmission spectrum of the PhCRR, where the resonance peaks (or the midpoint positions of split resonances) are marked with asterisks [106].

Gao et al. developed an air-mode PhCRR on a silicon-on-insulator platform, where the optical mode is strongly confined within the PhC air holes [106]. This confinement was confirmed through three-dimensional FDTD simulations, and the combination of whispering-gallery resonance with the slow-light effect in the PhC waveguide enhances light–matter interaction. The simulated and measured spectra show nonuniform free spectral ranges near the Brillouin-zone edge, indicating slow-light dispersion. In this region, the device achieves a group index of 27.3 and a quality factor of 14,600, demonstrating strong potential for refractive-index sensing, integrated emitters, and nonlinear photonics. Figure 6a presents the SEM image of the fabricated PhCRR. Light is coupled using a narrowed strip waveguide to improve phase matching and spatial overlap, with a 170 nm coupling gap and a 375 nm waveguide width. All structural parameters follow the simulation design. The measured transmission spectrum in Figure 6b shows resonances from 1530 to 1560 nm, including mode splitting due to coupling between counter-propagating modes. As the wavelength approaches the PhC band edge, the free spectral range decreases from 3.38 to 0.86 nm, confirming strong dispersion and slow-light behavior. This slow-light enhancement increases the resonator Q factor, reaching 12,100 at 1558.6 nm and peaking at 14,600 near 1555.8 nm, which is advantageous for sensing, light-emission enhancement, and nonlinear-optical applications [106].

### 4.3. Slot PhCWs

Slot PhCWs combine photonic bandgap confinement with a nanoscale low-index channel embedded in the defect region. This configuration significantly enhances the electric field intensity inside the slot because the discontinuity in dielectric constant forces a redistribution of the field toward the low-index region [7]. As a result, slot PhCWs provide exceptional interaction between the guided mode and the analyte, yielding sensitivity levels that surpass those of conventional defect guided modes [107]. The slot geometry increases the available design space by enabling independent control of slot width, hole arrangement, and defect symmetry. These degrees of freedom allow targeted tailoring of both the distribution field and the local dispersion landscape. For sensing applications, this enables structures in which the modal energy is concentrated almost entirely within the slot, thereby maximizing the contribution of analyte-induced refractive index changes to the overall wavelength shift [108]. However, slot-based architectures require fabrication precision on the order of a few nanometers. Variations in slot width or interface roughness significantly affect the mode confinement and the attainable propagation length. Furthermore, the combination of strong confinement and engineered dispersion demands careful fabrication control to avoid unwanted scattering and maintain spectral stability. Despite these challenges, slot PhCWs remain among the most promising configurations for ultra-high sensitivity detection.

Jannesari et al. introduced the design and simulation of a highly responsive sensor built on a slot PhCW [7]. The device was formed from a dielectric slab patterned with a triangular lattice of cylindrical air holes, which were subsequently filled with SiO_2_. A waveguide channel was created by removing a line of lattice elements and incorporating a central slot. This configuration significantly boosts the proportion of the evanescent field, resulting in strong interaction between the guided mode and the surrounding analyte. It was demonstrated that, for a fixed slab thickness, the slot PhCW exhibits a substantial improvement in sensitivity up to 7.6 times higher than that of conventional PhCW structures or silicon slab waveguides. To analyze the waveguide’s dispersion characteristics and mode profiles, both finite-difference time-domain (FDTD) simulations and the plane-wave expansion (PWE) method were employed [7].

Furthermore, Gao et al. reported the design, fabrication, and experimental evaluation of a suspended slotted photonic crystal (SSPhC) cavity sensor implemented on a silicon-on-insulator platform [7]. By carefully optimizing the SSPhC cavity, a substantial increase in sensing performance was achieved, as a large fraction of the optical field, approximately 57%, was concentrated in the low-index slot region. Monitoring the spectral response of the device when exposed to NaCl solutions of varying concentrations allows us to demonstrate an exceptionally high sensitivity of about 656 nm/RIU. In addition, the complete footprint of the sensor, including its grating couplers, measures only 320 × 40 µm^2^, making this high-performance architecture promising for scalable, multi-channel on-chip sensing systems [7].

### 4.4. Hybrid and Novel Platforms

Hybrid device architectures extend the capabilities of PhCWs by incorporating materials or structural elements beyond conventional silicon-based lattices [109,110]. Polymer silicon hybrid structures introduce functional materials that offer greater chemical compatibility, tunable refractive index, or improved thermal stability. By coating or partially filling PhC holes with polymers, it is possible to create devices with enhanced surface interaction properties and reduced thermooptic drift compared with fully silicon structures [111,112]. Another emerging direction involves integrating two-dimensional materials such as graphene or molybdenum disulfide. These atomically thin layers introduce strong light-matter coupling, electro-optical tunability, and surface adsorption characteristics that complement the inherent dispersion engineering capabilities of PhCs. When positioned in regions of high optical intensity, these materials can enable active modulation, selective biochemical interaction, or hybrid refractive index sensing.

Plasmonic PhC platforms represent an additional class of hybrid architectures where metallic elements are introduced near the defect channel [113]. This combines the long-range guiding and low-loss transport of dielectric PhCs with the intense localized fields characteristic of plasmonic nanostructures. Although metallic components introduce absorption, when carefully engineered, they provide strong localized enhancement that can be beneficial for specific sensing modalities requiring extreme field concentration. Accurate refractive index measurement is essential in bio-optical diagnostics. Khani et al. introduced two sensor configurations designed for cancer cell detection [114]. Both designs used one-dimensional PhC lattices coupled to metal–insulator–metal plasmonic waveguides, with a tapered interface added in the second configuration to improve coupling. The PhC region generated well-defined band gaps that produce steep spectral transitions suitable for sensing. Because the two bandgap edges were widely separated, shifts in the lower edge caused by analyte refractive index changes do not overlap with the upper edge, enabling operation over a broad wavelength range. The proposed structures achieved sensitivities of 718.6 and 714.3 nm/RIU and FOM of 156.217 and 60.1 RIU^−1^. Silver, air, and gallium arsenide form the material platform. Device performance was analyzed using finite difference time domain simulations, supported by transmission line modeling. The compact layouts, along with high spectral responsiveness, make these sensors strong candidates for biosensing applications [114].

Opto-mechanical photonic crystals represent an emerging extension of PhC-based sensing, where optical modes are coupled to engineered mechanical resonances [115]. In these systems, environmental perturbations such as refractive-index variation, mass loading, or pressure induce measurable shifts in mechanical or optical spectra through optomechanical interaction. The combination of slow-light enhancement and strong optomechanical coupling enables highly sensitive, multi-parameter detection within compact integrated platforms, expanding the functionality of conventional PhCW sensors [115].

These hybrid and novel platforms broaden the functionality of PhCWs beyond purely passive sensing. They support active control, enhanced chemical specificity, and multimodal operation that extends sensing capabilities into regimes not achievable with dielectric structures alone [116]. Wang et al. presented a theoretically investigated surface-enhanced Raman scattering sensor that achieved extremely large local field amplification through the coupling of surface plasmon polaritons [117]. The device employed a hybrid metal–dielectric slot waveguide formed by thin gold layers combined with a silicon nitride strip. In this configuration, the silicon nitride section was not used to carry the excitation mode; instead, it acts as a supporting layer that strengthens field confinement within the nanoscale slot. This approach concentrated optical energy more effectively than purely metallic or purely dielectric slot geometries. Simulations demonstrated that the structure could generate field enhancement factors greater than 10^6^ using a compact footprint of approximately 510 × 300 × 225 nm^3^ at an excitation wavelength of 785 nm. Parameter studies confirmed that the geometry can be readily adapted for Raman spectroscopic sensing and is compatible with micro and nano photonic integration for on-chip detection applications [117].

**Figure 7:**
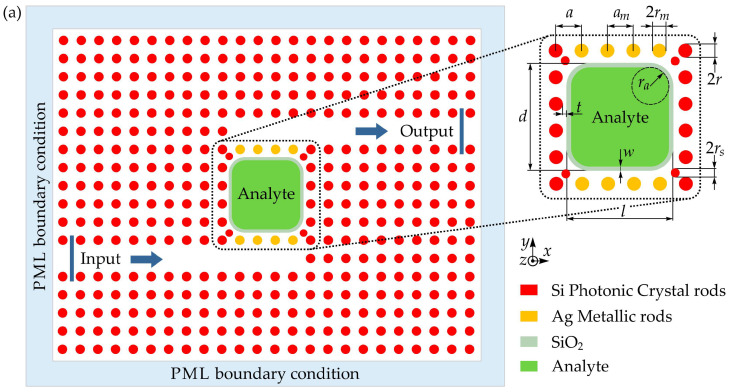

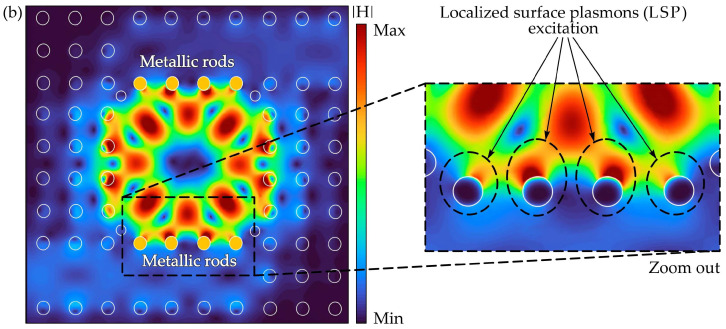
(**a**) Two-dimensional schematic of the proposed hybrid P–PhC refractive-index sensor; (**b**) magnetic-field intensity distribution (|H|) of the hybrid P–PhC sensor at the resonance wavelength of 1860 nm [118].

Hajshahvaladi et al. presented the design of a refractive-index (RI) sensor that combined plasmonic and PhC mechanisms within a hybrid P–PhC architecture. The device incorporates metallic rods into a rod-type PhC lattice, enabling the excitation of localized surface plasmons (LSPs) within the periodic structure (Figure 7a). Finite-difference time-domain (FDTD) simulations were used to analyze the sensor’s optical response. To better illustrate the operating principle of the device, Figure 7b presents the magnetic-field distribution (|H|) of the hybrid sensor at its resonant wavelength of 1860 nm. In the main schematic, the metallic rods were indicated with yellow circles, whereas the enlarged view omits these markers so the interaction between the field and the metal regions can be observed clearly. The field pattern shows that the metallic rods located in the coupling sections successfully excite localized surface plasmon modes within the PhC lattice. As highlighted in the magnified region of Figure 7b, the electromagnetic field becomes strongly concentrated along the metal–dielectric boundary and extends into the analyte-filled cavity. By contrast, in the silicon rods, the field primarily occupies the rod interior. This behavior demonstrates that the combination of plasmonic excitation in the metal rods and guided modes in the PhC structure gives rise to a hybrid P–PhC mode, which enhances the device’s sensitivity and improves its spectral resolution.

The results show that the resulting hybrid plasmonic–photonic mode enhances light–matter interaction, particularly when metallic rods are positioned in the coupling regions between the waveguides and the cavity. This configuration amplifies the optical field within the sensing region and strengthens its interaction with the analyte. The simulated performance demonstrates a sensitivity of 1672 nm/RIU and FOM of 2388 RIU^−1^, values that surpass those of previously reported sensors based solely on plasmonic components or conventional PhC structures. The ability to simultaneously achieve high sensitivity and a large FOM positions the proposed hybrid P–PhC sensor as a strong candidate for applications requiring precise, high-resolution refractive-index detection at optical communication wavelengths [118].

## 5. Applications

The practical relevance of PhCW sensors arises from their ability to convert refractive-index variations into measurable optical signatures with high precision. Because their dispersion characteristics can be tailored to enhance light–matter interaction without fundamentally altering the device footprint, PhCWs can be adapted to a wide variety of sensing environments, including biological fluids, chemical mixtures, and particulate suspensions [119]. Their compatibility with integrated photonic platforms further supports implementation in compact, robust, and application-specific systems. The subsections below outline the main domains where these capabilities translate into meaningful performance advantages.

### 5.1. Biochemical Sensing

PhCWs are particularly effective in detecting biochemical interactions occurring at or near functionalized surfaces [120]. In biological assays involving proteins, nucleic acids, or other biomolecules, binding events cause nanoscale refractive-index perturbations that the guided mode can detect with high fidelity due to its strong evanescent penetration into the sensing region. The engineered dispersion of PhCWs enables stable spectral responses even when monitoring weak binding affinities or low analyte concentrations. Importantly, their planar geometry supports seamless integration with microfluidic sample handling, allowing controlled delivery of reagents and minimizing sample volumes. This combination of high sensitivity, compactness, and flow-compatible integration makes PhCWs suitable for applications such as medical diagnostics, biomarker screening, and real-time monitoring of biochemical kinetics [121].

Gonzalez et al. analyzed a highly sensitive interferometric PhC sensor designed to determine glucose levels in human urine samples [63]. The simulated device was structured as a Mach–Zehnder interferometer that incorporated a waveguide–cavity coupling configuration. Its operation relied on photonic mode transitions observed through photonic band diagram analysis. These mode transitions were triggered by variations in glucose concentration, which modify the refractive index within the sensing cavity. A shift in the photonic mode produces a phase difference between the signal traveling through the reference arm and the electromagnetic wave propagating inside the sample-loaded cavity. This phase shift altered the output transmittance, enabling the sensor to respond sharply to refractive index changes. Because the sensor output was governed by transmittance variations, a sensitivity metric was defined based on the change in transmittance relative to a unit change in refractive index. The PhC sensor exhibited a sensitivity of 7000%/RIU, corresponding to a 70% transmittance change for a refractive index variation of 0.01. These findings highlight the potential of mode-transition–based mechanisms for achieving high-performance sensing in integrated photonic platforms [63].

**Figure 8:**
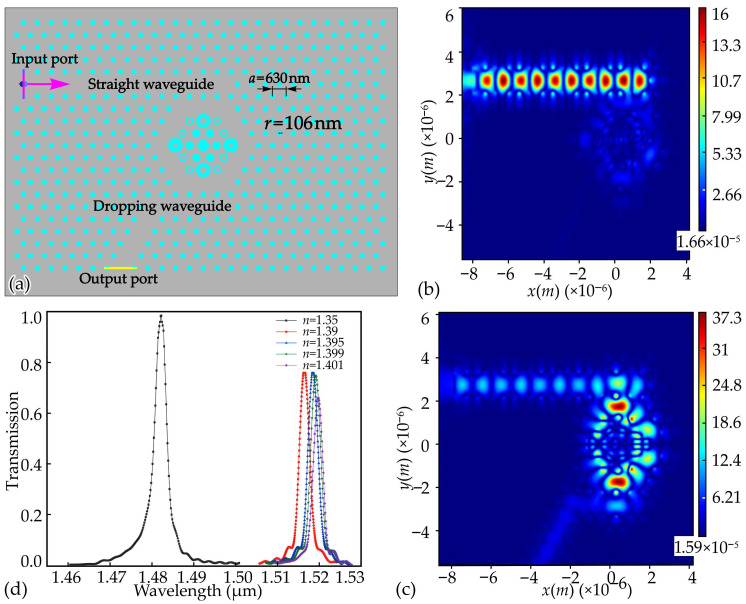
(**a**) Schematic representation of the two-dimensional PhC biosensor; (**b**) optical power distribution of the structure at a wavelength of 1455 nm; (**c**) optical power distribution of the structure at a wavelength of 1469 nm; (**d**) transmission spectra of the proposed biosensor for various normal and cancerous cell samples [122].

Recently, Yang et al. introduced a cancer-cell-sensing device based on a silicon PhC that incorporated a hexagonal resonant cavity within a triangular lattice arrangement (Figure 8a) [122]. The proposed design exhibited a photonic bandgap in the wavelength region of 1188–1968 nm. When illuminated with light in this interval, the device shows a transmission peak of 99.62% at a resonant wavelength near 1469.58 nm, as determined through finite-difference time-domain simulations, and demonstrates a quality factor of 980 [122]. Using this configuration, a biosensing platform was proposed whose operation depends on resonance shifts caused by changes in the refractive index of the sample. By monitoring the movement of the resonant wavelength, different biological targets can be distinguished. Since cancerous and healthy cells feature distinct refractive indices, the sensor could differentiate between them. Figure 8b and Figure 8c illustrate the electric field profiles of the proposed sensor under OFF-resonance and ON-resonance conditions, respectively. Figure 8d provides the transmission spectrum for the sample S1, offering a clearer view of how the biosensor differentiates normal and cancerous cells. As can be observed, any modification in the dielectric column’s refractive index leads to a measurable wavelength shift. The device achieved a maximum sensitivity of 915.75 nm/RIU and a minimum detectable refractive-index variation of 0.000236 RIU [122].

### 5.2. Environmental and Chemical Sensing

In chemical and environmental monitoring, PhCW sensors provide reliable detection of refractive-index variations associated with concentration changes or the presence of specific dissolved or airborne species [123,124]. Because the optical mode can be engineered to overlap strongly with infiltrated liquids or gases, the sensor maintains high responsiveness even when analyte concentrations are low. This enables precise measurement of parameters such as salinity, pollutant levels, and solvent composition [125,126]. The inherent stability of SOI-based PhCWs also allows operation in diverse environmental conditions, supporting field monitoring and industrial process control [127]. When integrated with on-chip microfluidics, the platform can perform continuous or in-line measurements without requiring bulk optical equipment, offering a pathway toward compact environmental sensing modules with high reliability [119,128,129,130].

A theoretical study was conducted to evaluate the sensitivity of an optical liquid sensor that utilizes a PhCW structure [60]. The sensing mechanism relied on changes in the analyte’s refractive index, which alter the effective index of the waveguide. A 3D finite element method was employed to model the sensor. The impact of various geometrical parameters on sensitivity was examined in detail. Findings indicate that carefully selecting these structural parameters allows the sensitivity to be significantly improved. Values exceeding 20 were obtained near the cut-off point in the slow-light regime, representing a substantial enhancement compared with traditional waveguide-based sensors [60].

The performance of MIR on-chip gas sensors based on laser absorption spectroscopy is largely determined by how effectively light interacts with the target molecules, which depends on both external confinement and the achievable optical path length. Conventional integrated sensors often struggle to reach the detection limits required for highly sensitive measurements because their effective light path is inherently short. In addition, standard silicon photonics platforms restrict the accessible MIR wavelength range. To address these challenges, Kim et al. proposed a new fabrication strategy for creating a freestanding germanium (Ge) PhCW on a germanium-on-insulator (Ge-OI) substrate that employs yttrium oxide (Y_2_O_3_) as the buried oxide layer. The wide transparency windows of both Ge and Y_2_O_3_ enable operation across a broader portion of the MIR spectrum. Incorporating the PhCW structure and exploiting its slow-light behavior substantially enhances light–matter interaction and external confinement, allowing the waveguide length to be significantly shortened, an important advantage for chip-scale sensing. The freestanding configuration further enlarges the sensing volume, improves confinement, and suppresses leaky modes within the PhCW. With these combined benefits, the resulting compact device attains a remarkably low limit of detection of 7.56 ppm for CO_2_ at 4.23 μm using a waveguide only 800 μm long [6].

### 5.3. Particle/Single-Molecule Sensing (Emerging)

PhCWs show growing potential in applications involving the detection of nanoscale particles and, in emerging cases, single molecular entities [131]. Their slow-light modes amplify scattering and perturbation effects from particles whose size falls well below the diffraction limit, enabling detection of entities such as viruses, extracellular vesicles, or engineered nanoparticles [132]. The spatial field distribution in PhCWs can be shaped to concentrate electromagnetic energy at specific lattice locations, improving the likelihood of interaction with transient particles carried by a fluid flow [131]. Although single-molecule sensing is not yet fully mature, advances in disorder mitigation, noise reduction, and cavity-enhanced PhCW configurations are closing this gap [133]. As these developments continue, PhCWs are expected to play a central role in next-generation platforms for nanoscale analytics and precision nanoparticle characterization [134].

A sensor for detecting biological molecules was developed using a thin, planar two-dimensional PhC membrane patterned with through-holes to confine strong optical fields [135]. This design enabled the detection of nanoparticles significantly smaller than the diffraction limit of a standard optical microscope. The fabrication of the PhC membrane was demonstrated, including the observation of a nanoparticle trapped within one of its holes. An imaging system combining a conventional optical microscope and a CCD camera was constructed to visualize the membrane, and the trapped nanoparticle appeared clearly as a bright spot. Initial experiments showed that the system could detect individual particles with radii down to approximately 75 nm.

**Figure 9:**
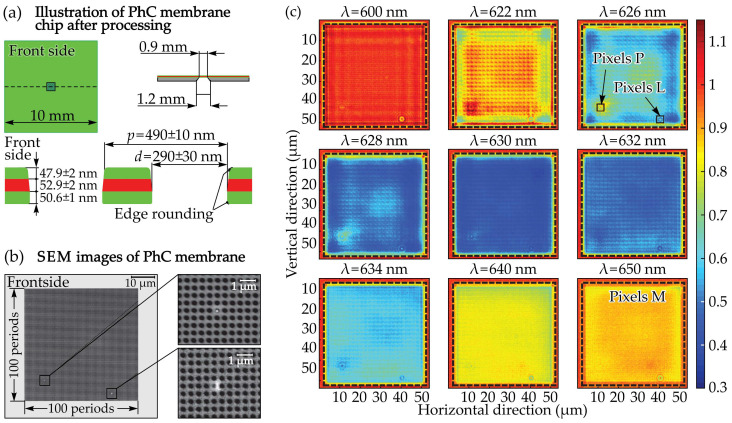
(**a**) Schematic representation of the processed PhC membrane chip; (**b**) top-view SEM images of the membrane, with enlarged views highlighting the two defect sites, (**c**) CCD images of the PhC membrane recorded at nine separate wavelengths. The dashed black outline marks the region corresponding to the hole array and identifies pixels labeled M. Pixels P and L indicate positions centered on the two defect sites within the membrane. The color scale represents the normalized pixel intensities, where each value is referenced to the transmission measured at the same wavelength when the membrane is removed [135].

The fabricated PhC membranes were released as free-standing structures at the center of 1 cm^2^ chips, with their geometry illustrated in Figure 9a. At a wavelength of 638.4 nm, the refractive indices of the constituent materials were measured to be 1.998 ± 0.01 for Si_3_N_4_ and 1.464 ± 0.01 for SiO_2_. No optical quality degradation due to mechanical stress was observed; tensile stress in the Si_3_N_4_ layer counteracts the compressive stress in the SiO_2_, maintaining membrane flatness. Slightly inclined hole walls and rounded edges, seen in Figure 9a, are inherent to the membrane release process. SEM images in Figure 9b present the final membrane and reveal two unintentional defects: a lattice irregularity extending approximately 2–3 periods vertically and 1.5 periods horizontally, and a nanoparticle with a radius of 75 ± 25 nm lodged in one of the holes. Although unintended, these features provide useful structures for initial characterization.

Figure 9c displays nine CCD images recorded at wavelengths ranging from 600 to 650 nm, spanning the guided resonance–associated transmission dip near λ = 630 nm. The large lattice defect remains visible across the full wavelength range, yet its appearance shows no clear dependence on resonance-related field enhancement. Instead, it is most distinct at 600 nm, where the internal field is expected to be low. This weak wavelength dependence, combined with its proximity to the membrane edge, indicates that the defect’s optical response is dominated by its geometry rather than resonance-enhanced scattering. In contrast, the nanoparticle exhibits a strong wavelength-dependent signal. At 600 nm it is barely visible due to low field strength and high background. As the wavelength approaches the guided resonance near 630 nm, the field within the membrane increases, and the nanoparticle becomes noticeably brighter. The resulting rise in contrast directly reflects the enhanced coupling of light into the resonance mode. Overall, the experiment demonstrates that the PhC membrane effectively amplifies the optical signature of nanoscale objects, enabling clear visual detection of particles well below the diffraction limit. The resonance-assisted field enhancement provides a reliable contrast mechanism, confirming the membrane’s suitability as a platform for sensitive nanoparticle and biomolecule detection [135].

## 6. Challenges and Practical Constraints

Although PhCWs offer exceptional opportunities for dispersion-engineered optical sensing, their practical performance is fundamentally shaped by fabrication imperfections, slow light-induced loss mechanisms, and environmental susceptibility. These constraints become increasingly significant as designs seek higher group indices, stronger modal confinement, and greater sensing overlap. The following subsections describe the dominant limitations that determine the operational boundaries of PhCW sensors.

### 6.1. Fabrication-Induced Disorder

The performance of slow-light PhCWs depends critically on the periodicity and precision of the underlying lattice. Nanometer-scale deviations in hole radius, lattice spacing, etch depth, or sidewall uniformity perturb the Bloch mode and introduce unwanted scattering. Because slow light modes possess large spatial Fourier components and interact strongly with the high contrast interfaces of the photonic crystal, even minor deviations from the intended geometry cause significant changes in the phase and amplitude of the guided mode. This scattering increases propagation loss and broadens spectral features, which directly worsens sensitivity and the limit of detection. Near the band edge, the mode can become partially localized, producing spatially inconsistent field distributions that reduce the predictability of the sensor response. The resulting spectral fluctuations cannot be eliminated through simple averaging because they arise from fixed structural imperfections rather than random measurement noise. Mitigation strategies rely on waveguide geometries that reduce field concentration at the boundaries of the holes, such as gentle confinement and dispersion-flattened designs. Inverse design and topology optimization provide additional routes for producing lattice perturbations that suppress sensitivity to dimensional variations [136]. Improvements in nanofabrication, including refined proximity correction and optimized etch chemistries, further reduce disorder-induced penalties by ensuring greater uniformity across the patterned region [31].

### 6.2. Loss and Sensitivity Interdependence

The strong light–matter interaction that enhances sensitivity in slow-light PhCWs also magnifies intrinsic and extrinsic loss mechanisms. As the group index increases, the propagation length effectively decreases due to enhanced sensitivity to both scattering and absorption. This behavior reflects the underlying physics of slow light transport, where reduced group velocity increases the interaction length with any perturbation, including fabrication imperfections and intrinsic material absorption. Although a higher group index improves the wavelength shift induced by refractive index changes, the corresponding increase in linewidth caused by elevated loss often reduces the achievable figure of merit [137]. For this reason, practical devices rarely operate at extremely high group index values predicted by idealized simulations. Instead, they are designed to maintain a moderate regime where the enhancement in interaction time still improves sensitivity without allowing loss to dominate the spectral response. Engineering approaches that balance overlap enhancement and attenuation control are therefore essential. Width-modulated and band-flattened PhCWs demonstrate improved overall sensing performance by enabling elevated modal overlap with only mild increases in group index. These structures preserve sensitivity improvements while preventing excessive line broadening [138].

### 6.3. Thermal Stability and Spectral Drift

The thermos-optic coefficient of silicon is high, and its influence becomes even more pronounced in slow-light PhCWs [139]. Temperature fluctuations shift the refractive index of the waveguide core and alter the position of the photonic band edge, causing measurable wavelength drift. Because the dispersion near the band edge is steep, even very small thermal changes produce disproportionately large spectral shifts, which significantly raise the noise equivalent wavelength shift and degrade detection resolution. Temperature gradients introduced by microfluidic flow, environmental heating, or localized absorption can further distort the mode profile along the waveguide [140]. These gradients lead to spatially varying refractive index distributions that cannot be easily compensated and that may shift the spectral response unpredictably during sensing.

To minimize thermal drift, researchers employ athermal designs that combine materials with opposite thermo-optic properties, modify lattice geometries to reduce band edge sensitivity, or incorporate claddings that counteract thermally induced index changes [141,142]. Silicon nitride and hybrid silicon polymer PhCWs offer naturally improved thermal stability due to their reduced refractive index temperature dependence [143,144]. Active stabilization using microheaters and feedback control remains another practical solution for maintaining consistent operating conditions, although it increases system complexity [145,146].

### 6.4. Packaging and Microfluidic Integration Constraints

Transitioning PhCWs from laboratory demonstrations to functional sensing modules requires effective packaging and fluid handling integration. Coupling light into submicron photonic crystal apertures demands precise alignment and mechanical stability [147]. Any misalignment or packaging-induced stress can distort the lattice or shift the effective refractive index, which alters the dispersion characteristics and undermines calibration. Microfluidic integration introduces additional complexity. The presence of channels or reservoirs above the photonic crystal modifies the cladding index and thus influences the guided mode [148]. Uniform analyte infiltration is crucial because inconsistent filling of the air holes changes the modal overlap and results in unstable sensitivity. Bubbles or particulate contaminants within the microchannel can temporarily perturb the optical mode, creating transient spectral fluctuations that complicate data interpretation. Chemical compatibility must also be ensured, particularly in applications involving aggressive solvents or biological reagents. Surface functionalization must remain stable over extended operation, and the boundary between the microfluidic environment and the photonic crystal should not degrade chemically or mechanically [149]. Ensuring long-term stability, therefore, requires careful material selection and robust bonding processes that maintain optical and mechanical integrity [150].

## 7. Future Directions

Future progress in PhCW sensors will depend on the ability to address the limitations that currently arise from fabrication imperfections, slow light propagation, and environmental sensitivity. As sensing applications demand higher accuracy, lower detection limits, and stable long-term operation, new design approaches and platform-level innovations will become increasingly important. Advances in computational design, material integration, and architectural refinement offer promising opportunities for improving device performance without compromising compactness or compatibility with integrated photonics. The following subsections outline the most significant research pathways that can guide the continued evolution of PhCW sensing technology.

### 7.1. Disorder-Resilient Slow-Light Designs

Fabrication-induced disorder remains one of the most significant barriers to achieving high performance in slow-light PhCWs [84]. Even minimal deviations in hole size or surface quality can increase scattering losses and broaden spectral responses, which reduces measurement precision. Future research is expected to focus on waveguide geometries that naturally minimize sensitivity to disorder while retaining large group indices. Examples include topologically guided modes, dispersion flattened structures, and gentle confinement methods that gradually modify the lattice. Improvements in fabrication processes, such as atomic layer smoothing and compensated pattern writing, can further reduce unwanted scattering. By combining robust physical design with advanced fabrication strategies, the next generation of devices may achieve strong slow light enhancement without compromising spectral stability, which is essential for reliable sensing performance [100,151].

Optical sensors that achieve a high FOM for refractive index detection can greatly improve sensing accuracy. In the case of guided-mode resonance (GMR) sensors, most prior studies have emphasized boosting sensitivity rather than optimizing the FOM, which has kept the best reported FOM values at around 100. To overcome this limitation, Zhou et al. introduced a symmetric GMR sensor fabricated from a low-index, UV-curable resin (*n* = 1.344) [152]. This design delivered higher sensitivity, a significantly narrower resonance linewidth, and a much larger FOM when operating in aqueous environments. A detailed analysis of the geometric parameters was performed, and numerical simulations predicted optimal FOM values reaching several tens of thousands. By employing a cost-effective nanoimprinting approach, a resonance linewidth of just 56 pm, a bulk refractive index sensitivity of 233.35 nm/RIU, and a detection limit of 1.93 × 10^−6^ were experimentally achieved. These results correspond to an FOM of 4200, which was about 48 times greater than that of typical GMR sensors. Owing to its stable, symmetric structure and high performance, the proposed GMR sensor is well-suited for a range of applications such as label-free biological detection, advanced imaging, and optical filtering [136].

### 7.2. Ultra-High Figure of Merit Sensors

Achieving high sensitivity and narrow linewidths at the same time remains difficult because slow light enhancement often increases optical loss [137]. One promising direction involves hybrid structures that combine the advantages of resonant cavities with those of PhCWs. These designs can provide strong field confinement while still allowing wide tunability and broad operating bandwidths [138]. Another approach involves coupling multiple resonant elements along a PhCW to sharpen the spectral response and increase the effective figure of merit. Multi-objective optimization using advanced dispersion engineering can also assist in achieving a balance between sensitivity, loss, and spectral resolution [139,140]. These strategies may enable devices that reach considerably higher figures of merit and therefore provide superior sensing capability during demanding measurement tasks [79].

### 7.3. Inverse-Designed PhCW Sensors

The design of PhCWs increasingly requires optimization methods that can navigate complex relationships among sensitivity, fabrication tolerance, and loss [141]. Inverse design techniques based on adjoint optimization and machine learning are well-suited for this purpose [142,143]. These methods can explore large design spaces and identify geometries that enhance modal overlap with the sensing region while reducing scattering from fabrication imperfections. Inverse-designed features such as optimized lattice perturbations, tailored cavity and waveguide coupling, and refined transitions can significantly improve overall device performance [144]. As computational tools continue to advance, inverse-designed PhCWs are expected to surpass conventional designs in both robustness and sensitivity, offering a powerful route toward next-generation integrated sensing systems [145].

### 7.4. Integrated Photonic Platforms

The integration of PhCWs with diverse material platforms is expected to expand their capabilities and support a wider range of sensing applications [146]. Silicon nitride provides lower propagation loss and reduced thermo-optic drift, which makes it suitable for environments that require high thermal stability [147,148]. Thin film lithium niobate offers electro-optic tunability that enables active control, rapid tuning, and dynamic calibration of the sensor [149,150]. Polymer-based structures and hybrid silicon devices incorporating two-dimensional materials can improve surface functionalization and increase the interaction strength with biochemical analytes [38,84]. The integration of PhCWs with on-chip microfluidics, light sources, and detectors will enable compact and self-contained sensing modules for clinical diagnostics, environmental monitoring, and real-time chemical analysis [153,154]. Nunes et al. presented an optofluidic platform that combined a silica-based one-dimensional PhC with integrated planar waveguides and electrically insulated microchannels [27]. Within the microfluidic path, a pillar array, originally intended for electrochromatographic separations, acts as an optical resonator enabling label-free, on-column refractive-index measurements. The device was realized using a silicon oxynitride material system, which supports electro-osmotic transport, and it was operated at a wavelength of 1550 nm. A series of ethanol–water mixtures with refractive indices between *n* = 1.3330 and 1.3616 was introduced into the resonant column, and the resulting transmission spectra were collected. Changes in refractive index produced linear shifts in the resonance dips, giving a maximum sensitivity of 480 nm/RIU. The system was capable of distinguishing refractive-index variations as small as 0.007 RIU [27]. These platform-level advancements will play an essential role in transitioning PhCW sensors from laboratory research toward practical and widely deployable technologies.

Beyond classical sensing applications, dispersion-engineered PhCWs exhibit features that intersect with emerging quantum photonic technologies [75]. The combination of slow-light enhancement, high-Q resonances, and strong light–matter interaction enables regimes relevant to single-photon nonlinearities, cavity quantum electrodynamics, and on-chip quantum light sources. In principle, dynamically controlled PhCW sensors operating at low photon numbers could approach quantum-limited detection, where sensitivity becomes constrained by shot noise rather than classical technical noise [155]. Furthermore, the ability to engineer dispersion and modal confinement with high precision positions PhCWs as promising platforms for integrated quantum photonic circuits, including photonic quantum computing architectures and quantum-enhanced sensing schemes. Although current implementations operate primarily in the classical regime, continued improvements in loss control, disorder mitigation, and active tuning may enable future devices to bridge classical slow-light sensing and quantum-limited measurement regimes [156].

## 8. Conclusions and Outlook

PhCWs represent one of the most powerful and versatile platforms for integrated optical sensing, owing to their inherently tailorable dispersion, strong light–matter interaction, and capacity for slow-light enhancement. This review has synthesized the fundamental physical principles, key performance metrics, device architectures, and application domains that define the current state of PhCW-based sensing. Collectively, the literature demonstrates that dispersion engineering remains the cornerstone of sensitivity improvement in PhCWs, but it simultaneously introduces practical constraints related to scattering loss, linewidth broadening, and disorder-induced instability. As a result, the true sensing performance of PhCWs is governed not by any single figure of merit but by the interplay among group index, modal overlap, spectral coherence, and fabrication tolerance. Across the reviewed studies, the most successful devices do not achieve the highest theoretical slow-light factors but a balanced operational regime that maximizes useful sensitivity while maintaining manageable loss and environmental stability.

This review also highlights the extensive diversity of architectural approaches that have been proposed to optimize this balance. Techniques such as gentle confinement, hole-position modulation, width perturbation, and slot integration have each demonstrated their ability to reshape dispersion, enhance analyte overlap, and suppress disorder sensitivity. Meanwhile, the emergence of hybrid material systems, including silicon nitride, polymers, two-dimensional materials, and plasmonic elements, expands the design space by introducing complementary optical, mechanical, and chemical properties. These developments indicate that the limitations traditionally associated with slow-light PhCs are increasingly tractable through informed design and materials engineering. At the same time, applications ranging from biochemical detection to environmental monitoring and nanoparticle analysis continue to push the requirements for detection limits, dynamic range, and operational robustness, underscoring the need for PhCW sensors that combine high performance with practical reliability.

Looking forward, several research directions appear particularly promising based on the collective trends identified in the literature. A major opportunity lies in the development of dispersion profiles that maintain high group indices while minimizing sensitivity to fabrication-induced disorder. Approaches such as inverse design, adjoint optimization, and topology-guided engineering are poised to play an increasingly central role in achieving such disorder-resilient slow-light structures. Another avenue for advancement involves hybrid and multi-functional PhCWs that integrate active materials, plasmonic enhancements, or electro-optic tuning to achieve dynamic control, improved chemical specificity, or enhanced signal contrast. Continued progress in microfluidic integration, packaging strategies, and thermal stabilization will also be essential for translating laboratory-scale demonstrations into practical, field-ready sensing modules.

Collectively, the literature reviewed in this work indicates a rapidly advancing field in which the inherent strengths of PhCWs, including precisely engineered dispersion, pronounced modal confinement, and high analyte–field overlap, are being enhanced through increasingly sophisticated design methodologies and integrated platform innovations. As nanofabrication capabilities continue to mature and computational optimization techniques gain broader adoption, PhCW sensors are expected to exhibit marked improvements in figure of merit, detection limit, and operational stability. The current trajectory of research strongly suggests that PhCWs will remain a foundational technology for next-generation integrated photonic sensing, offering a robust combination of high sensitivity, scalability, and structural tunability suitable for a wide spectrum of emerging sensing applications.

## Figures and Tables

**Table 1:** Fundamental comparison between PhCWs and other integrated waveguide types for optical sensing.

Waveguide Structure	Confinement Mechanism	Dispersion Engineering	Sensitivity Potential	Key Advantages	Key Limitations	References
Photonic Crystal Waveguide (PhCW)	Photonic bandgap + defect-guided mode	Excellent (hole shifting, width modulation, gentle confinement)	High: strong slow-light enhancement, increased interaction time, large effective index change per perturbation	Strong slow-light enhancement; high analyte overlap; precise dispersion control; broadband sensing	Sensitive to fabrication disorder; n_g_–loss trade-off	[6,18,25,32]
Strip/Rib Waveguide	Total internal reflection (TIR)	Limited (geometry-based only)	Moderate: moderate evanescent-field fraction; sensitivity scales linearly with mode overlap	Robust, low-loss, CMOS-friendly	Weak mode–analyte interaction; minimal dispersion tunability	[33,34,35,36,37]
Slot Waveguide	TIR with nanoslot field enhancement	Moderate	Very High: extremely strong electric-field density in the slot → high modal index perturbation	Extremely strong field overlap with analyte; high refractive-index sensitivity	High fabrication sensitivity; increased scattering loss	[38,39,40]
Subwavelength Grating (SWG) Waveguide	Bloch-mode index modulation (1D periodic)	Moderate	Moderate–High: enhanced field penetration; engineered effective index	Tailorable effective index; relaxed fabrication tolerance	Limited slow-light enhancement compared to PhCW	[41,42,43,44,45]
Nanobeam Waveguide (non-cavity)	Index contrast waveguiding	Moderate	Moderate: improved field localization relative to the strip; limited slow-light capability	Compact footprint; moderate modal overlap	Weaker bandgap effects than 2D PhCWs	[46,47,48]

**Table 2:** Comparative and interdependent behavior of key performance metrics in slow-light PhCW sensors.

Metric	Primary Determinants	Positive Influences	Negative Influences/Degradation Mechanisms	Interdependencies with Other Metrics	Overall Implications for PhCW Design
Sensitivity (S) [62,64,84]	Group index (n_g_), modal overlap with analyte, dispersion slope near band edge	Slow-light enhancement increases wavelength shift per RIU; higher field penetration into the sensing region; strong analyte-mode overlap	Disorder-induced scattering increases spectral noise; modal localization reduces repeatability; thermal fluctuations inflate baseline drift	Increased S → increased linewidth noise → reduced FOM; high S often correlates with poorer LOD due to increased noise; excessive n_g_ reduces dynamic range linearity	Theoretical S increases with n_g_, but effective sensitivity is capped by noise and disorder; optimal S occurs at moderate n_g_ regimes
Figure of Merit (FOM = S/FWHM) [85,86,87]	Sensitivity, spectral linewidth, propagation loss, disorder sensitivity	Mild slow-light operation improves S while maintaining narrow linewidths; gentle confinement and band-flattening reduce GVD and scattering	Linewidth broadening from slow-light loss; scattering and vertical radiation leakage; high disorder sensitivity at large n_g_	FOM drops sharply when FWHM grows faster than S; designs maximizing S may lower FOM if linewidth increases proportionally or superlinearly	FOM has a distinct optimum—not maximized by the highest n_g_ but by balanced dispersion engineering, minimizing loss and GVD
Limit of Detection (LOD) [88,89]	Noise-equivalent wavelength shift (NEWS), thermal stability, laser stability, spectral linewidth	Lower spectral noise via reduced GVD; temperature-stable materials or hybrid claddings; narrow linewidth via low-loss slow-light designs	Temperature drift increases NEWS; laser jitter impacting spectral resolution; disorder-induced mode wandering; high *n_g_* intensifies all noise sources	High S does not guarantee low LOD; LOD correlates more with linewidth stability and thermal noise than raw S; increased FWHM → worse LOD	Best-case LOD requires suppressing environmental noise more than boosting S; LOD is often the practical bottleneck in slow-light sensing
Dynamic Range (DR) [90]	Dispersion linearity, band-edge proximity, spectral shift saturation	Operation away from steep band-edge regions; flattened dispersion reduces nonlinear response; moderate *n_g_* ensures quasi-linear regime	Deep slow light induces nonlinear wavelength shift vs. Δ*n*; GVD causes nonuniform sensor response; saturation near cutoff reduces usable sensing range	Designs maximizing S typically minimize DR due to increased nonlinearity; DR competes directly with slow-light enhancement providing high S	DR is reduced by aggressive slow-light engineering; practical devices require a balance between linearity and sensitivity for broad analyte ranges
Group Index (*n_g_*) (underlying driver) [70,91]	Lattice engineering, hole shifting, dispersion flattening	Higher light–matter interaction → increased S; enables compact, high-performance sensors	Scattering loss ∝ *n_g_*^2^– *n_g_*^3^; Linewidth broadening reduces FOM; thermal drift amplified; nonlinearity near band-edge reduces DR	Affects S, FOM, LOD, DR simultaneously; no metric scales independently with *n_g_*	*n_g_* is the central trade-off parameter: beneficial for S but detrimental for FOM, LOD, and DR beyond a threshold

**Table 3:** Comparison of major PhCW architectures and dispersion-engineering strategies.

Architecture	Main Mechanism	Strengths	Limitations	Notes/Typical Use
Conventional W1 PhCW [92,93]	Line defect in a periodic lattice; bandgap guiding	Simple design, CMOS-compatible, predictable dispersion	Limited slow-light bandwidth; higher GVD near band edge	Baseline architecture for sensing and dispersion studies
Hole-shifted PhCW [94,95]	Longitudinal or lateral displacement of holes	Flatter dispersion, reduced GVD, moderate high *ng*	More sensitive to fabrication variations	Good for broadband slow-light sensing
Gentle-confinement PhCW [96]	Gradual lattice perturbation to reduce scattering	Low loss, spectrally stable, improved slow-light bandwidth	Requires careful optimization of hole radii/positions	Ideal for low-loss high-FOM sensing
Width-modulated PhCW [97,98]	Variation in waveguide width to tune dispersion	Wide flat-band slow-light regions; disorder-resilient	Complex design and fabrication	Effective for stable high-*ng* performance
Slot-integrated PhCW [99]	Field concentration in a sub-wavelength slot	Extremely high modal overlap; very high sensitivity	High fabrication tolerance; narrow slot critical	Best for biochemical and surface-binding sensors
Hybrid/2D-material PhCW [100,101]	Integration of graphene, MoS_2_, polymers, liquids	Enables tunability, enhanced analyte interaction	Added absorption, integration complexity	Expands sensing modalities; thermal stability improvements

## Data Availability

Not applicable.
